# Synthesis and CO_2_ Capture of Porous Hydrogel Particles Consisting of Hyperbranched Poly(amidoamine)s

**DOI:** 10.3390/gels8080500

**Published:** 2022-08-11

**Authors:** Hojung Choi, Sanghwa Lee, SeongUk Jeong, Yeon Ki Hong, Sang Youl Kim

**Affiliations:** 1 Department of Chemistry, Korea Advanced Institute of Science and Technology (KAIST), Daejeon 34141, Korea; 2 School of Chemical and Materials Engineering, Korea National University of Transportation, Chungju-si 27469, Korea

**Keywords:** carbon dioxide capture, poly(amidoamine)s, hyperbranched polymers, macroporous polymers, suspension polymerization

## Abstract

We successfully synthesized new macroporous hydrogel particles consisting of hyperbranched poly(amidoamine)s (HPAMAM) using the Oil-in-Water-in-Oil (O/W/O) suspension polymerization method at both the 50 mL flask scale and the 5 L reactor scale. The pore sizes and particle sizes were easily tuned by controlling the agitation speeds during the polymerization reaction. Since O/W/O suspension polymerization gives porous architecture to the microparticles, synthesized hydrogel particles having abundant amine groups inside polymers exhibited a high CO_2_ absorption capacity (104 mg/g) and a fast absorption rate in a packed-column test.

## 1. Introduction

A major anthropogenic greenhouse gas in the atmosphere, CO_2_ gas has been emitted in large quantities, mainly due to the burning of fossil fuels and other chemical processes [1,2]. Despite the global economic slowdown caused by the COVID-19 pandemic, the atmospheric concentration of CO_2_ increased to 421 ppm in May 2022, from 405 ppm in 2017 and 340 ppm in the 1980s [3,4], and the estimated amount of annual CO_2_ capture necessary to maintain the current level of atmospheric CO_2_ concentrations has reached 3.7 gigatonnes/year [5]. However, fossil fuels are the major source of energy for human activities [6,7], and this trend is expected to continue over the coming decades [8]. Therefore, capture and storage of CO_2_ gas from power plants is an urgent and important issue with respect to reducing CO_2_ emissions.

The current CO_2_ capture technology from flue gases involves the passage of streams of CO_2_-containing flue gases through an aqueous amine solution, for example, monoethanolamine (MEA), in the absorber column. The amine solution is then regenerated by heating it in a stripping tower to liberate the CO_2_. This ‘wet-scrubbing’ method with an alkanolamine solution has been employed industrially since the 1980s and is suitable for large-scale removal of CO_2_ [8]. However, the regeneration of the total solution requires considerable energy consumption, and the amine concentration of the solution is limited due to viscosity and foaming [9]. Furthermore, careful control of the alkanolamine solution is necessary to avoid corrosion and oxidative degradation [2,10].

In efforts to overcome these problems, recently many materials have been developed as dry type solid CO_2_ adsorbents. In the early stage of research, porous inorganic materials drew interest as solid type adsorbents for their high surface area [11,12,13]. However, industrial flue gases contain a considerable amount of water vapor [14], and these types of adsorbents are vulnerable to moisture, resulting in diminution of their sorption capacity and durability [15,16]. To address these drawbacks, amine-containing CO_2_ adsorbents that tolerate moisture have been developed, including amine-modified mesoporous silica [17,18,19,20,21,22,23,24,25], Metal-Organic Frameworks (MOFs) [26,27,28,29,30,31], and amine-containing polymeric adsorbents [32,33,34,35,36]. However, implementing amines in solid supports disrupts the pore structure and reduces the surface area [25,37,38]. Furthermore, nonuniform dispersion of the amine and the complicated synthetic process of amine-implemented solid CO_2_ adsorbents are hurdles toward practical applications. 

**Scheme 1:**
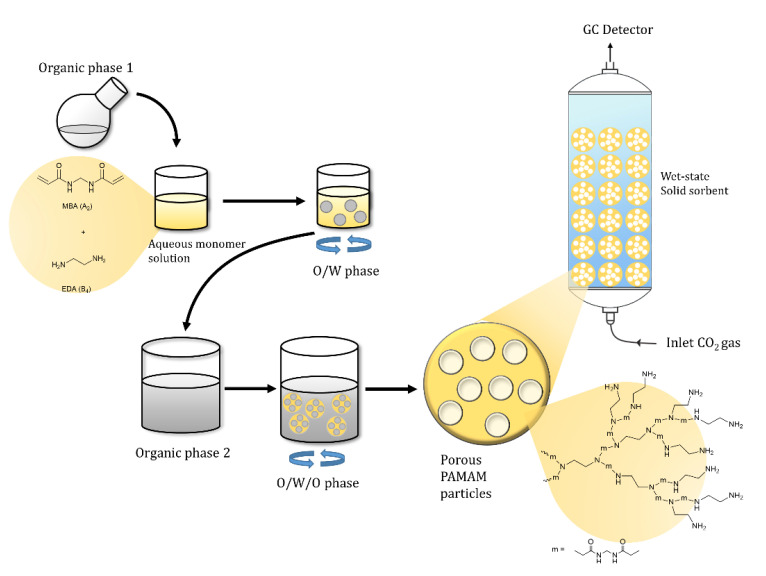
Schematic illustration of synthesis and carbon dioxide capture of porous HPAMAM.

To exploit the strengths of wet-scrubbing and a dry type adsorbent, we designed a hydrogel absorbent that works in an aqueous phase. A hydrogel is introduced because hydrogels are widely used as adsorbents for various substances such as heavy metal ions, dyes, and carbon dioxide due to their high adsorption performance and ease of use in an aqueous solution [39,40,41,42,43,44,45,46]. Because amines are verified carbon dioxide absorbers in wet-scrubbing processes by forming carbamate and ammonium bicarbonate with CO_2_, we adopted poly(amidoamine) (PAMAM) as our key functional material. PAMAM has a high content of N-heteroatom groups. Abundant secondary and hindered amine groups of PAMAM are expected to provide high CO_2_ sorption capacity [10,47]. Previously, we reported a facile one-step synthesis and application of micro-sized hyperbranched PAMAM (HPAMAM) particles [48]. Compared with PAMAM dendrimers, HPAMAM particles offer advantages of simplicity in the synthetic process and ease of use as packed columns without an ultrafiltration step for separation. In this work, we synthesize porous HPAMAM particles and investigate the CO_2_ sorption ability of the porous HPAMAM hydrogel microbeads in packed columns (Scheme 1). The amine groups of HPAMAM particles in the packed column resemble the environment of amines in conventional wet scrubbing. Emulsion templating including double emulsions is generally used to produce porous materials. We adopted the Oil-in-Water-in-Oil (O/W/O) suspension polymerization method to provide a porous feature to HPAMAM particles that resembles the structural pores of solid type adsorbents [49,50,51,52]. The macropores inside of the hydrogel particles facilitate mass transfer and diffusion and thereby accelerate the sorption rate.

## 2. Results and Discussion

The preparation of porous HPAMAM particles was carried out via the O/W/O suspension polymerization method. We prepared three solutions: (1) an inner phase organic solution (O_1_, solution 1) used to create interior pores; (2) an aqueous solution (W, solution 2) in which a polymerization reaction actually takes place; and (3) an outer phase organic solution (O_2_, solution 3) for developing spherical particles.

A_2_ (N,N’-methylenebisacrylamide, MBA) and B_4_ (ethylenediamine, EDA) monomers and surfactant (poly(vinylalcohol), PVA) were mixed together in aqueous solution W until complete dissolution of the solids to induce aza-michael addition between amine and acrylamide. The O_1_ solution, containing a cosurfactant (Span 60^®^) and toluene, was added to aqueous solution W with vigorous agitation to make an O/W suspension. The O/W mixture was then suspended to the O_2_ solution. Since the polymerization of hydrophilic monomers occurs in the aqueous phase, the gelated polymer forms a spherical shape with the inner dispersion of organic solutions. After the polymerization, during the workup, the inner organic phase was removed from the particles, leaving macropores. Samples of porous HPAMAM particles are designated as **P-MxAyTz**, where x, y and z denote the molar feed ratio of MBA to EDA, the agitation speed (rpm) of the O/W suspension, and the volume ratio of solution 1 to solution 2, respectively (Scheme 1).

**Figure 1:**
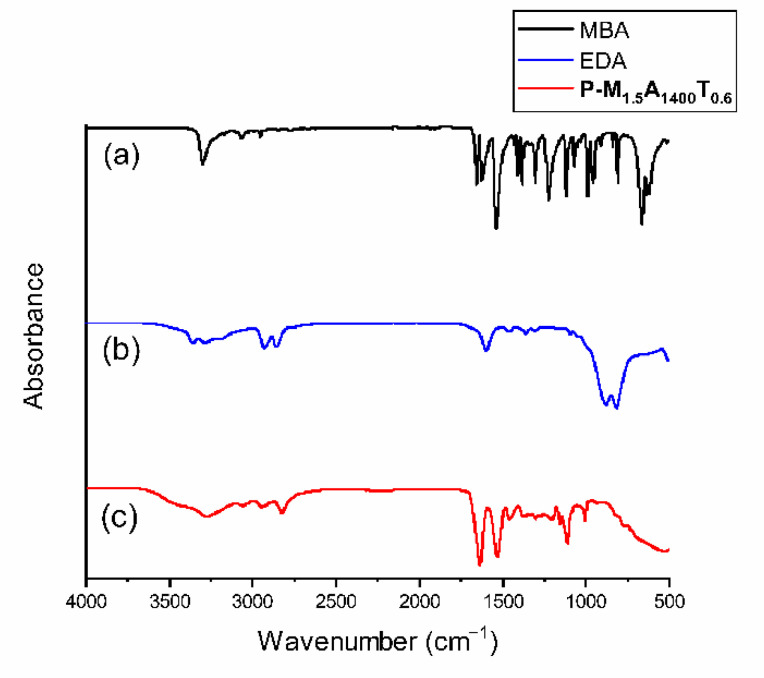
ATR-FTIR spectra of methylenebiscrylamide (a), ethylenediamine (b), and poly(amidoamine) particles (**P-M_1_._5_A_1400_T_0_._6_**) (c).

**Figure 2:**
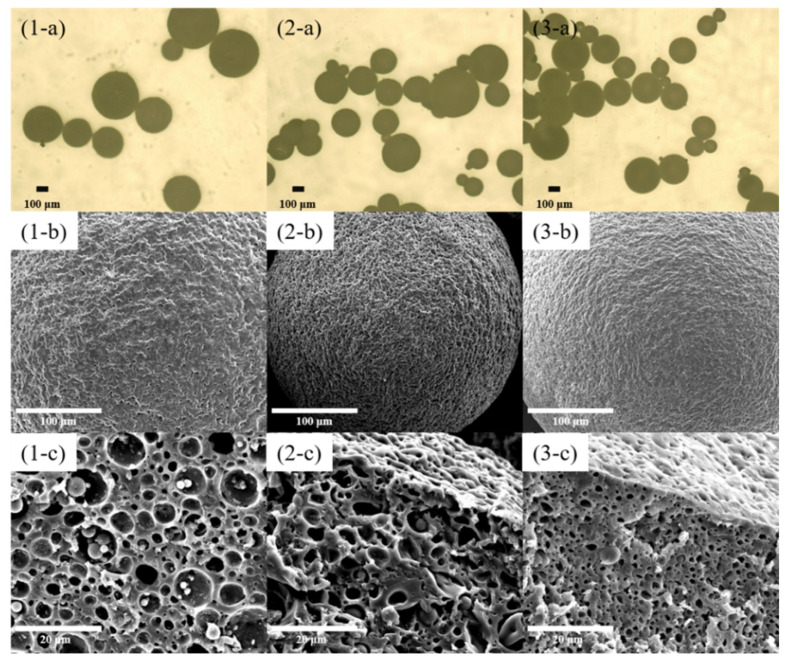
Optical images (**a**), SEM image (**b**), and cross-sectional SEM image of (**1-a**, **1-b** and **1-c**) **P-M_1_._5_A_1400_T_0_._6_**, (**2-a**, **2-b** and **2-c**) **P-M_1_._5_A_3000_T_0_._6_**, and (**3-a**, **3-b** and **3-c**) **P-M_1_._5_A_6000_T_0_._6_**. Magnification is ×1000 (**b**) and ×5000 (**c**).

**Figure 3:**
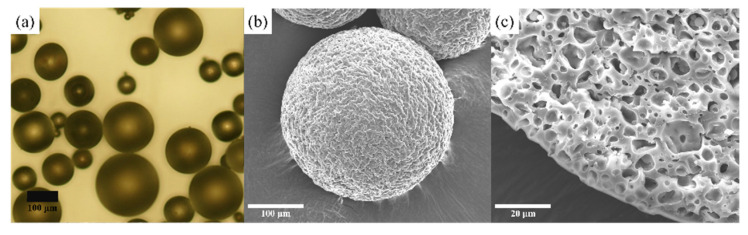
Optical images of **P-M_1_._5_A_1400_T_0_._6_-2L** (**a**) and cross-sectional SEM images of **P-M_1_._5_A_1400_T_0_._6_-2L** (**b**) ×1000 and (**c**) ×5000.

Three kinds of porous HPAMAM particles, **P-M_1_._5_A_1400_T_0_._6_**, **P-M_1_._5_A_3000_T_0_._6_**, and **P-M_1_._5_A_6000_T_0_._6,_** were synthesized via O/W/O suspension polymerization with different agitation speeds to verify that the pore size can be controlled by the agitation speed of the O/W phase. To verify the chemical structures of the product, we carried out ATR-FTIR analysis for a representative sample of **P-M_1_._5_A_1400_T_0_._6_** (Figure 1). ATR-FTIR spectra of MBA, EDA, and PAMAM polymers showed the peaks of N-H stretching (3000~3350 cm^−1^), C=O stretching (1650 cm^−1^), and CONH stretching (1520 cm^−1^) in the PAMAM product, suggesting the successful synthesis of poly(amidoamine). The C=CH_2_ stretching peak (3020 cm^−1^) of MBA disappeared in the PAMAM product. The 3080 cm^−1^ peak is an overtone of 1540 cm^−1^ (N-H bending), which commonly appears in amide compounds. Energy Dispersive X-ray Spectroscopy (EDS) was performed to obtain additional information on the monomer ratio (Figure S1). The morphology of the synthesized porous HPAMAM particles was investigated with an optical microscope (OM) and a scanning electron microscope (SEM), as shown in Figure 2. Particle sizes obtained with a slower agitation speed (1000 rpm) were smaller than the particle sizes obtained at a faster agitation speed (1400 rpm) (Figure S2). Since the size of the pores was in the range of the micrometer scale, we analyzed the pore sizes through SEM images rather than with porosimetry such as BET, which is suitable for micro or mesopores. The average pore size changed from 2.9 μm to 2.3 μm to 1.3 μm with each agitation speed of 1400 rpm, 3000 rpm, and 6000 rpm, respectively (Figure 2c and Figure S3). As expected, the pore size decreased with the increase of the O/W phase agitation speed. However, the size of the particles did not change regardless of the O/W phase agitation speed (50–300 μm). The proposed synthetic route for porous HPAMAM hydrogel particles via O/W/O suspension polymerization is an eco-friendly process. It does not produce any byproducts and does not require catalysts or pore-generating reagents (porogens). Scale-up synthesis of the porous HPAMAM hydrogel particles was attempted with O/W/O suspension polymerization. The 2 L scale O/W/O suspension polymerization reaction of MBA and EDA monomers proceeded successfully and a product (**P-M_1_._5_A_1400_T_0_._6_-2L**) with 50–300 μm particle diameters was obtained (Figure 3). The porous HPAMAM hydrogel particles exhibited no differences compared to the particles obtained with the smaller scale reaction. The synthesized porous PAMAM hydrogel particles contain a large amount of amine functional groups that are known to interact with carbon dioxide to form carbamates (Figure S4). On the basis of the feasibility of large-scale production through an eco-friendly process and the high reactivity of the amines in synthesized polymers to carbon dioxide [10,47,53,54], the porous HPAMAM hydrogel particles are suitable CO_2_ absorbing materials for flue gas.

**Figure 4:**
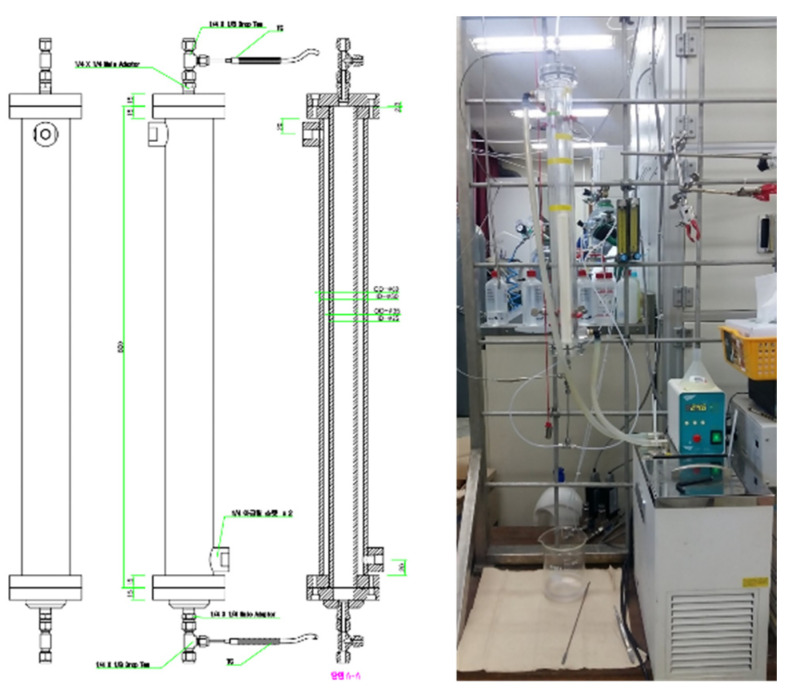
Schematic illustration of the column and a photograph of the experimental setup.

**Figure 5:**
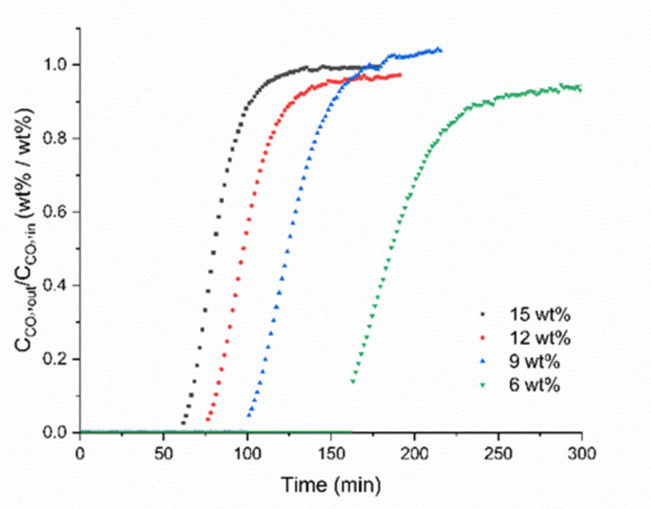
CO_2_ sorption properties of porous PAMAM hydrogel particles (**P-M_1_._5_A_1400_T_0_._6_-2L**) with various CO_2_ concentrations of input gas.

To measure the CO_2_ absorption capacity of the hydrogel particles, water-soaked porous HPAMAM particles (**P-M_1_._5_A_1400_T_0_._6_-2L**) packed in a 60 cm column were used with a continuous flow of CO_2_. A schematic illustration of the column and a photograph of the experimental setup are shown in Figure 4. We used a column with a pocket where water can be circulated to control the temperature of the column at 50 °C. For the measurement, 45 g of porous PAMAM particles (**P-M_1_._5_A_1400_T_0_._6_-2L**) was used at various CO_2_ concentrations (6, 9, 12, and 15 wt%) with a gas flow rate of 150 mL/min (Figure 5). The concentration of outlet CO_2_ gas was measured by gas chromatography at 25 °C. The porous HPAMAM hydrogel particles exhibited 2.374 mol/kg of CO_2_ sorption (104 mg/g) within 60 min for saturation at 15 wt% CO_2_ concentration while the sorption value was 2.150 mol/kg and 100 min for saturation at 9 wt% CO_2_. These values are comparable to the reported values of amine-modified mesoporous silica (1.36–2.59 mol/kg) [35,55,56].

## 3. Conclusions

We synthesized a new type of CO_2_ absorbing porous hydrogel particle consisting of hyperbranched poly(amidoamine)s by Oil-in-Water-in-Oil (O/W/O) suspension polymerization of N,N’-methylenebisacrylamide (MBA) and ethylenediamine (EDA) monomers. By introducing the double emulsion method to the conventional inverse suspension polymerization, synthesis of macroporous hydrogel particles was achieved successfully. The particle sizes and the size of macropores were controlled by simply changing the agitation speed of Oil-in-Water-in-Oil (O/W/O) suspension polymerization, demonstrating the high applicability of the synthetic method in a large scale. The synthesized porous PAMAM hydrogel particles contain many amine functional groups that are known to interact with carbon dioxide. A CO_2_ sorption test revealed the CO_2_ absorption capacity of the porous hydrogel particles was 104.4 mg/g, which is comparable to the values of high performance dry-state adsorbents. Easy scale-up of the O/W/O suspension polymerization of MBA and EDA without any byproducts supports the practical usability of porous hydrogel particles for CO_2_ capture from flue gas.

## 4. Materials and Methods

### 4.1. General Information

Ethylenediamine (EDA), N,N’-methylenebisacrylamide (MBA), poly(vinylalcohol) (PVA) (Mw = 89,000–98,000 g/mol), and Span 60^®^ were purchased from Sigma-Aldrich (Boston, MA, USA). Methanol (MeOH), toluene, and cyclohexane were purchased from Junsei (Chuo-ku, Tokyo, Japan). All reagents were used as received without further purification. A 5L Chemglass ChemRxnHub (Vineland, NJ, USA) Process system was used in the scale up process. Images of the particles were obtained by Nikon ECLIPSE ME600 (Tokyo, Japan) optical microscopy (OM), FEI Nova230 (Hillsboro, OR, USA), and JEOL JSM IT800 (Akishima, Tokyo, Japan) scanning electron microscopy (SEM). EDS analysis was conducted by a Bruker Quantax ESPRIT (Billerica, MA, USA) attachment. CO_2_ was measured by DS6200 gas chromatography (DS Science Inc.; Gwangju-si, Gyeonggi-do, Korea).

### 4.2. Various Agitation Speeds of Aqueous Phase

Three solutions, solution 1 (Span60 (0.128 g, 5 wt%) in toluene (3 mL)), solution 2 (MBA (2.024 g), EDA (0.526 g), PVA (0.026 g, 1 wt%) in water (5.1 mL)), and solution 3 (Span60 (0.013 g, 0.5 wt%) in toluene (20.4 mL)) were prepared. Solution 3 was stirred vigorously at 60 °C. MBA, EDA, PVA, and water in solution 2 were mixed together until complete dissolution of the solids at 45 °C. After solution 1 was added to solution 2, it was agitated at 1400 rpm (stirring bar), 3000 rpm (homogenizer), and 6000 rpm (homogenizer) for one minute. After that, solution 2 was added to solution 3, and agitated at 1400 rpm at 60 °C. After 6 hr of polymerization, particles were filtered and washed with methanol and acetone, and dried under vacuum at 70 °C for 24 hr.

### 4.3. Scale up Process

Three solutions, solution 1 (Span60 (25 g, 0.5 wt%) in toluene (600 mL)), solution 2 (MBA (398.6 g), EDA (103.2 g), PVA (5 g, 1 wt%) in water (1 L)), and solution 3 (Span60 (1.5 g, 0.5 wt%) in toluene (2 L)) were prepared. Solution 3 was stirred vigorously at 60 °C. MBA, EDA, PVA, and water in solution 2 were mixed together until dissolution of the solids at 45 °C. After solution 1 was added to solution 2, it was agitated at 5000 rpm (homogenizer), for one minute. Solution 2 was then added to solution 3, and it was agitated at 1400 rpm at 60 °C. After 6 hr of polymerization, particles were filtered and washed with methanol and acetone, and dried under vacuum at 70 °C for 24 h.

### 4.4. CO_2_ Absorption Test

Water was poured to 45 g of each type of PAMAM particle (P1.5, P1.8, P2.0, and P1.5 porosity) in a beaker. After 90 min, wet PAMAM particles were placed in an acryl column (diameter: 7 cm, height 60 cm). Removing the moisture in column, N_2_ gas was flowed for 20 min. CO_2_ absorption was then carried out using 15 wt%, 12 wt%, 9 wt% and 6 wt% of CO_2_ (The rest of the gas was N_2_) at a flow rate of 150 mL/min at 50 °C. The concentration of CO_2_ in the outlet gas was measured by gas chromatography.

## Data Availability

The data presented in this study are available on request from the corresponding author.

## Supplementary Materials

The following supporting information can be downloaded at: https://www.mdpi.com/article/10.3390/gels8080500/s1, Figure S1: SEM and EDS analysis of P-M_1_._5_A_1400_T_0_._6_; Figure S2: SEM images of P-M_1_._5_A_3000_, W/O agitation speed of 1400 rpm (a,b) and P-M1.5A3000, W/O agitation speed of 1000 rpm (c,d); Figure S3: Pore size distribution of various O1/W agitation speeds, 1400 rpm (a), 3000 rpm (b), 6000 rpm (c), and merged graph (d), based on SEM images of Figure 2; Figure S4: Reactions of amine and carbon dioxide [1,2,3,4].
