# Supplementary material for: Synthesis and CO2 Capture of Porous Hydrogel Particles Consisting of Hyperbranched Poly(amidoamine)s
Source: Gels. 2022 Aug 11;8(8):500. doi: 10.3390/gels8080500 (PMC9407192; doi:10.3390/gels8080500)
Supplement: Supplementary file 1 [file gels-08-00500-s001.zip › gels-1842202-supplementary.pdf]

Supplementary Materials

Synthesis and CO_2 Capture of Porous Hydrogel Particles Consisting of Hyperbranched Poly(amidoamine)s

Hojung Choi [1,†], Sanghwa Lee [1,†], SeongUk Jeong [1], Yeon Ki Hong [2] and Sang Youl Kim [1,*]

[1] Department of Chemistry, Korea Advanced Institute of Science and Technology (KAIST), Daejeon 34141, Korea
[2] School of Chemical and Materials Engineering, Korea National University of Transportation, Chungju-si 27469, Korea
[*] Correspondence: kimsy@kaist.ac.kr; Tel.: +82-42-350-2834
[†] These authors contributed equally to this work.

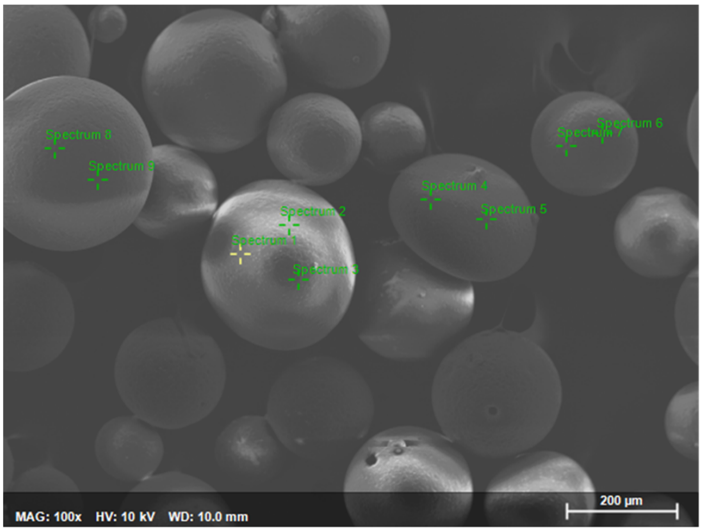

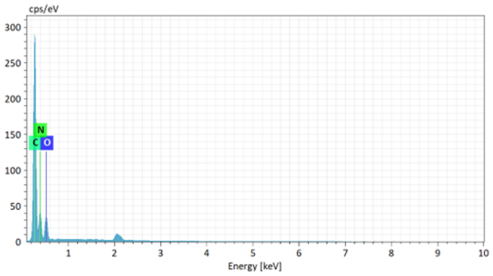

EDS, wt%	C	O	N
Average	50.94	18.55	30.51
Error (%)	0.61	1.52	1.41

Figure S1. SEM images and EDS analysis of **P-M$_{1.4}$A$_{1400}$T$_{0.6}$**.

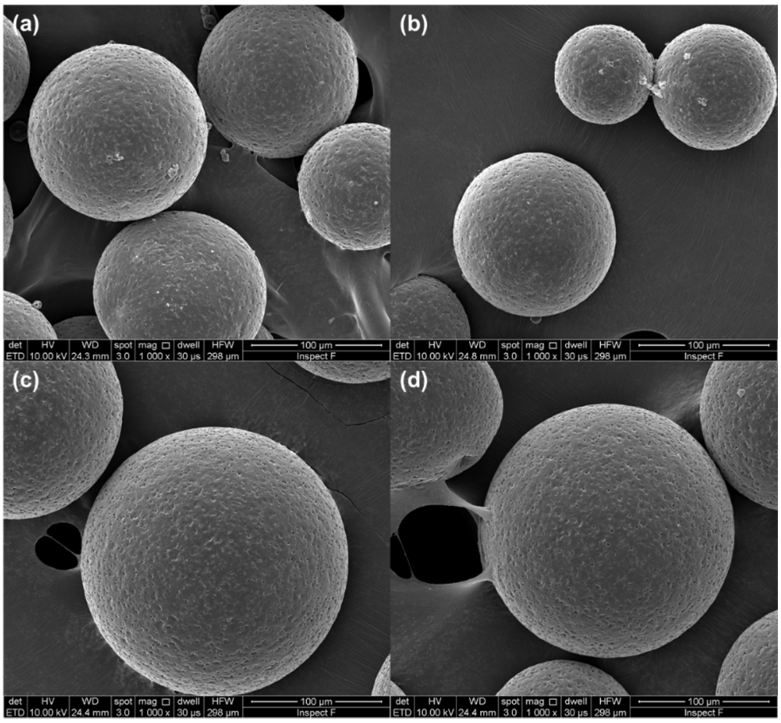

Figure S2. SEM images of **P-M1.5A3000**, W/O agitation speed of 1400 rpm (**a**,**b**) and P-M1.5A3000, W/O agitation speed of 1000 rpm (**c**,**d**).

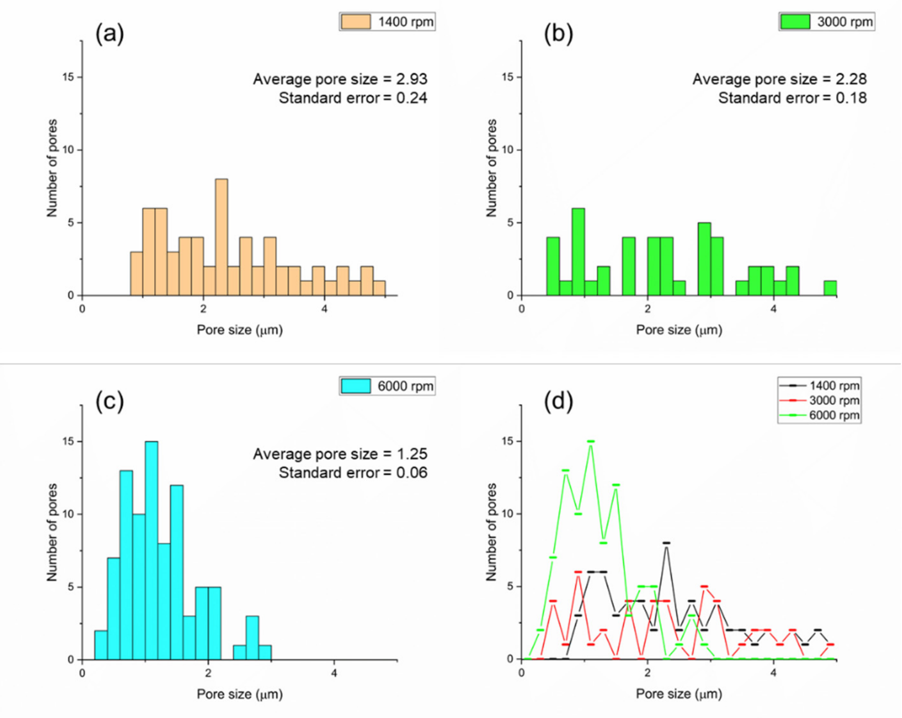

Figure S3. Pore size distribution of various O1/W agitation speeds, 1400 rpm (**a**), 3000 rpm (**b**), 6000 rpm (**c**), and merged graph (**d**), based on SEM images of Figure 2.

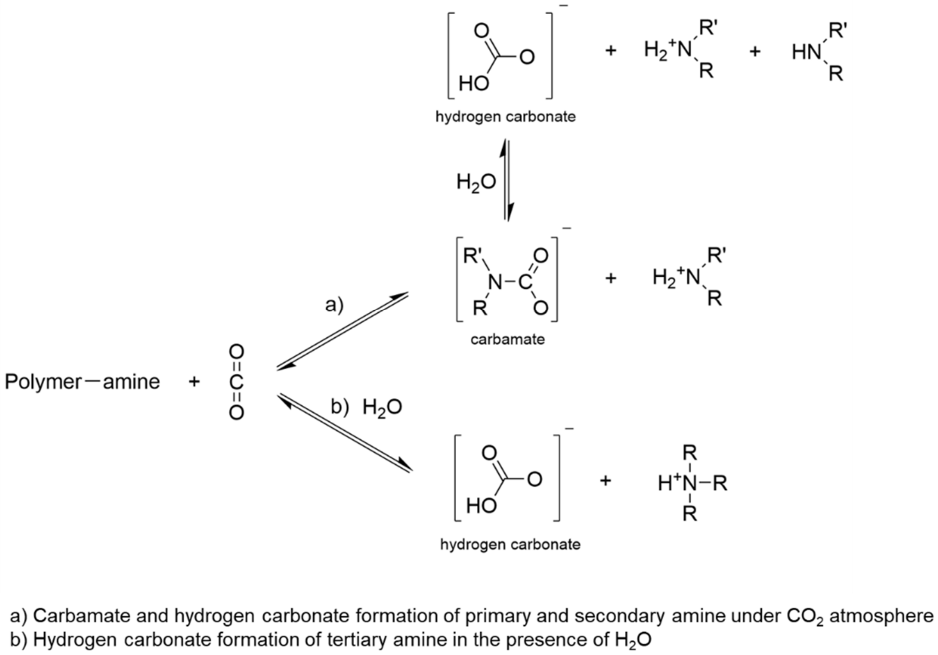

Figure S4. Reactions of amine and carbon dioxide [1–4].

References

1. D'Alessandro, D.M.; Smit, B.; Long, J.R. Carbon Dioxide Capture: Prospects for New Materials. *Angew. Chem. Int. Ed.* **2010**, *49*, 6058–6082.
2. Henao, W.; Jaramillo, L.Y.; López, D.; Romero-Sáez, M.; Buitrago-Sierra, R. Insights into the CO_2 capture over amine-functionalized mesoporous silica adsorbents derived from rice husk ash. *J. Environ. Chem. Eng.* **2020**, *8*, 104362.
3. Taniguchi, I.; Kinugasa, K.; Toyoda, M.; Minezaki, K. Effect of amine structure on CO_2 capture by polymeric membranes. *Sci.*

Technol. Adv. Mater. **2017**, *18*, 950–958.
4. Varghese, A.M.; Karanikolos, G.N. CO_2 capture adsorbents functionalized by amine-bearing polymers: A review. *Int. J. Greenh. Gas Control* **2020**, *96*, 103005.
